# Osteogenic cocktail induces calcifications in human breast cancer cell line via placental alkaline phosphatase expression

**DOI:** 10.1038/s41598-020-69622-7

**Published:** 2020-07-29

**Authors:** Atsushi Fushimi, Hiroshi Takeyama, Toshiaki Tachibana, Yoshinobu Manome

**Affiliations:** 1 Core Research Facilities, The Jikei University School of Medicine, 3-25-8, Nishi-shimbashi, Minato-ku, Tokyo, Japan; 2 Department of Surgery, The Jikei University School of Medicine, Tokyo, Japan

**Keywords:** Breast cancer, Breast cancer

## Abstract

Breast cancer is frequently characterized by calcifications in mammography. The mechanism for calcifications in breast cancer is not completely known. Understanding this mechanism will improve diagnostic accuracy. Herein, we demonstrated that calcifications occur and that alkaline phosphatase enzyme activity increases in MDA-MB-231 cells cultured using an osteogenic cocktail-containing medium. Microarray transcript analysis showed that the PI3K-Akt signaling pathway was significantly involved, with recruitment of placental alkaline phosphatase. Calcifications and alkaline phosphatase enzyme activity were suppressed by silencing placental alkaline phosphatase using a small interfering RNA. Inhibition of the PI3K-Akt signaling pathway suppressed phospho-c-Jun and placental alkaline phosphatase and resulted in absence of calcifications. These findings reveal that breast cancer cells acquire alkaline phosphatase enzyme activity via placental alkaline phosphatase expression and suggest that breast calcification formation is closely associated with the PI3K-Akt signaling pathway.

## Introduction

Breast microcalcifications are an important finding on mammography. A systematic review of clinical studies beginning before 1990 showed that screening mammography reduced mortality in women aged 40 to 69 years^1^ by 15% to 20%. An article on the history of mammography noted that Leborgne reported in 1949 that about 30% of breast cancers had calcifications on X-ray^2^. Low-voltage mammography devised by Egan in 1959 became the foundation for current mammography^2^. Leborgne’s diagram described the characteristics of breast cancer calcifications as “scattering of countless, punctiform or elongated calcifications, very closely grouped, particularly in center”^2^. Benign and malignant calcifications can be discriminated by their shape. Guidelines include classifications based on the morphology and distribution of calcifications^3,4^. However, in cases in which it is difficult to distinguish benign from malignant calcifications based on morphology and distribution, additional investigations such as biopsy or magnetic resonance imaging are required. This limits the ability to make a diagnosis based on calcifications alone. In fact, it is not known how breast cancer calcifications are produced. If this mechanism were understood, it might be possible to improve the accuracy of diagnosis by calcifications on mammography by combining imaging with some other marker such as a blood test related to that underlying mechanism.

Although breast calcifications were previously thought to be produced passively as the final stage of cell regression, recent studies have shown active production^5^. In another context, vascular smooth muscle cells have been found to produce vascular calcifications by acquiring osteoblast-like characteristics^6^. The same mechanism has been suggested to account for breast calcifications. Cox et al. showed that breast calcifications are clearly generated by the mouse breast cancer cell line 4T1 through osteoblast-like characteristics^7^^,^ the details underlying such a mechanism of mammary calcification is unknown.

We induced calcifications using the human breast cancer MDA-MB-231 cell line. Our hypothesis was that breast cancer calcifications are generated in a process similar to that of osteoblast differentiation. By a comprehensive analysis of transcription compared with osteoblastic cell line MC3T3-E1 in a microarray, we showed that placental alkaline phosphatase (PLAP) is involved in producing calcifications through PI3K-Akt signaling pathways.

## Results

**Figure 1:**
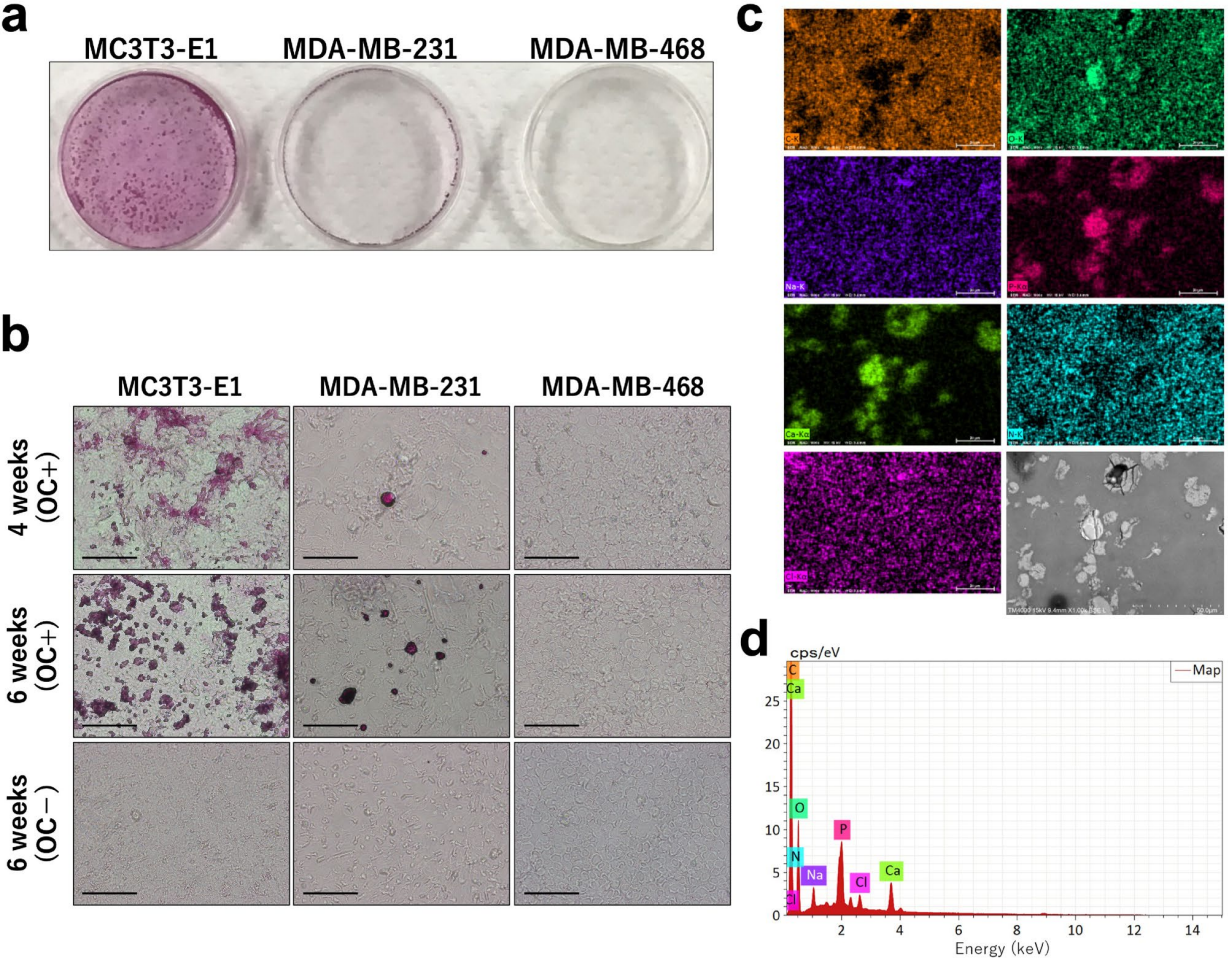
MDA-MB-231 cells produced calcifications that are rich in calcium and phosphorous. (**a**) Alizarin staining shows calcifications in MC3T3-E1, MDA-MB-231, and MDA-MB-468 cells cultured with an osteogenic cocktail (OC) for 6 weeks. (**b**) Cells cultured with OC for 4 weeks and with or without OC for 6 weeks (magnification × 20, scale bar 10 µm). (**c**,**d**) Elemental analysis of MDA-MB-231 cells; HITACHI TM4000 HV 15.0 keV 1,000 × WD 9.4 mm. The mass of each element is C: 55.09%, N: 6.14%, O: 29.32%, Na: 1.27, P: 3.63%, Cl: 0.93%, and Ca: 3.62%.

Human breast cancer cell lines MDA-MB-231and MDA-MB-468 and mouse preosteoblast cell line MC3T3-E1 were cultured in medium containing an osteogenic cocktail (OC). Alizarin staining showed formation of calcifications in 4 weeks in MDA-MB-231 cells, and the calcifications were diffusely spread by 6 weeks (Fig. 1a,b). Compared with MC3T3-E1 cells, MDA-MB-231 cells started to produce calcifications later and less abundantly. MDA-MB-468 cells did not produce calcifications until 6 weeks. We used MDA-MB-468 cells as a negative control in this experiment. The compound produced by MC3T3-E1 cells is calcium phosphate^8^^,^ which is what is often clinically observed in breast cancer^9^. Elemental analysis of MDA-MB-231 cells indicated they were rich in calcium and phosphorous, confirming that the calcifications produced by MDA-MB-231 cells in this experiment were calcium phosphate (Fig. 1c).

**Figure 2:**
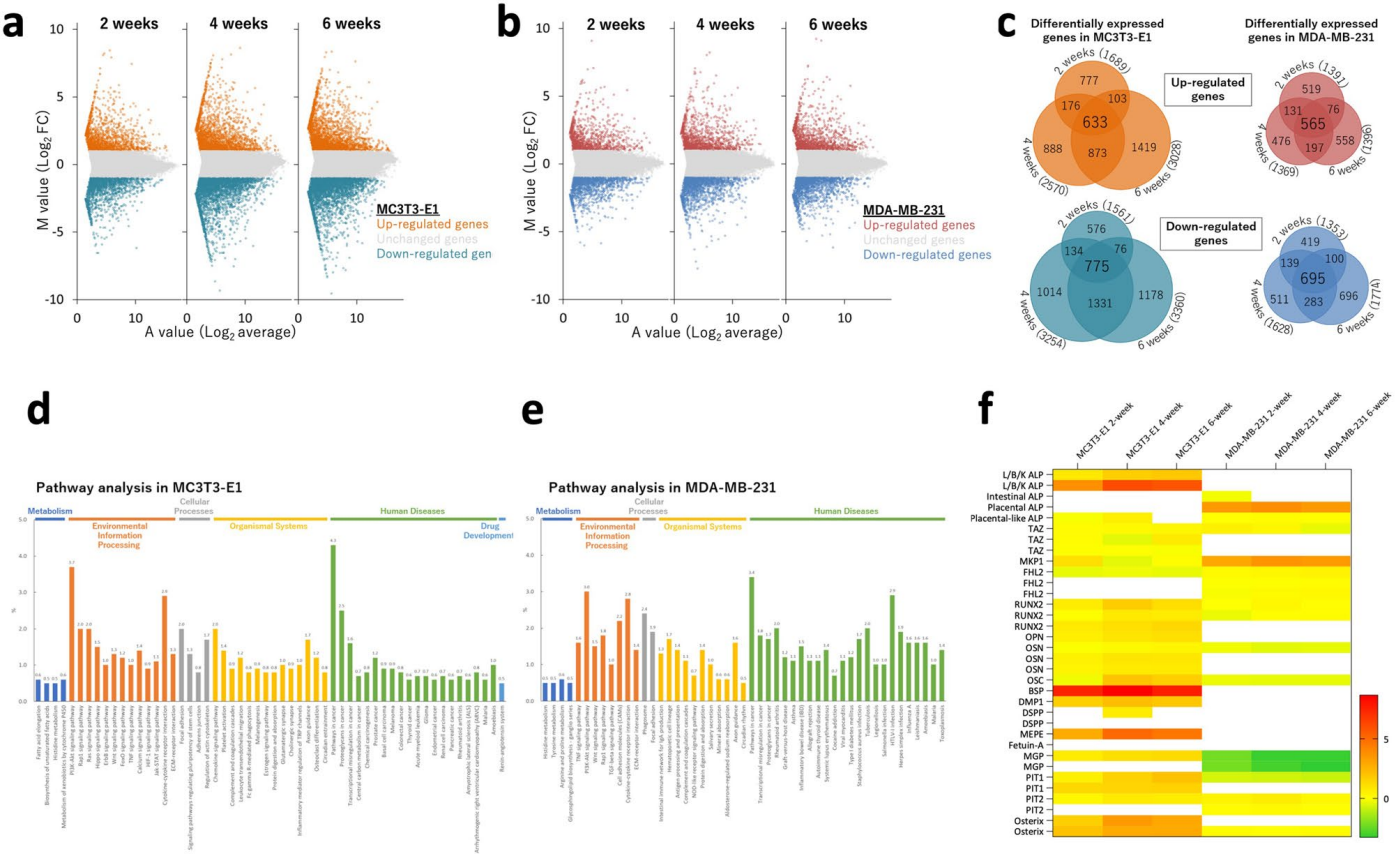
Microarray analysis shows the difference in differentially expressed genes between MC3T3-E1 and MDA-MB-231 cells cultured with or without an osteogenic cocktail MA plots of (**a**) MC3T3-E1 and (**b**) MDA-MB-231 cells. (**c**) Diagram of differentially expressed genes after 2, 4, and 6 weeks of culture. Pathway analysis of (**d**) MC3T3-E1 and (**e**) MDA-MB-231 cells. (**f**) Bone-related gene expressions on MC3T3E1 and MDA-MB-231 cells.

Microarray analysis was performed to explore biological differences between MDA-MB-231 and MC3T3-E1 with regard to formation of calcifications. MA plots showed genes that were differentially expressed by administering OC to MC3T3-E1 and MDA-MB-231 cells at weeks 2, 4, and 6 (Fig. 2a,b). In MC3T3-E1 cells, 633 genes were upregulated and 775 were downregulated. In MDA-MB-231 cells, 565 genes were upregulated and 695 were downregulated (Fig. 2c). There were therefore 1,408 differentially expressed genes in MC3T3-E1 and 1,260 in MDA-MB-231. Pathway analysis using KEGG pathway indicated high frequencies of *PI3K-Akt signaling* and *pathways in cancer* in both MDA-MB-231 and MC3T3-E1 cells, suggesting a relationship between these pathways and formation of calcifications (Fig. 2d,e). Gene set enrichment analysis revealed that the mTOR signaling pathway factors tend to increase in MDA-MB-231 cells cultured using OC (NES: 1.77, p-value: 0.076, q-value: 0.099) and that PI3K-AKT signaling in cancer is associated with the formation of calcifications (NES: 1.46, p-value: 0.047, q-value: 0.169) (Supplementary Fig. 1).

Microarray analysis data were used to see if there was a difference in mRNA expression between MC3T3-E1 and MDA-MB-231 cells for reported bone-related genes. There were more bone-related genes with increased expression in MC3T3-E1 cells and fewer in MDA-MB-231 cells (Fig. 2f). Although *ALPL*, a gene for tissue-nonspecific alkaline phosphatase (TNAP), was upregulated in MC3T3-E1 cells, it was not detected in MDA-MB-231 cells. Instead, *ALPP,* a gene for placental alkaline phosphatase, was upregulated in MDA-MB-231 cells. This suggests that PLAP may be involved in the formation of calcifications in MDA-MB-231 cells.

**Figure 3:**
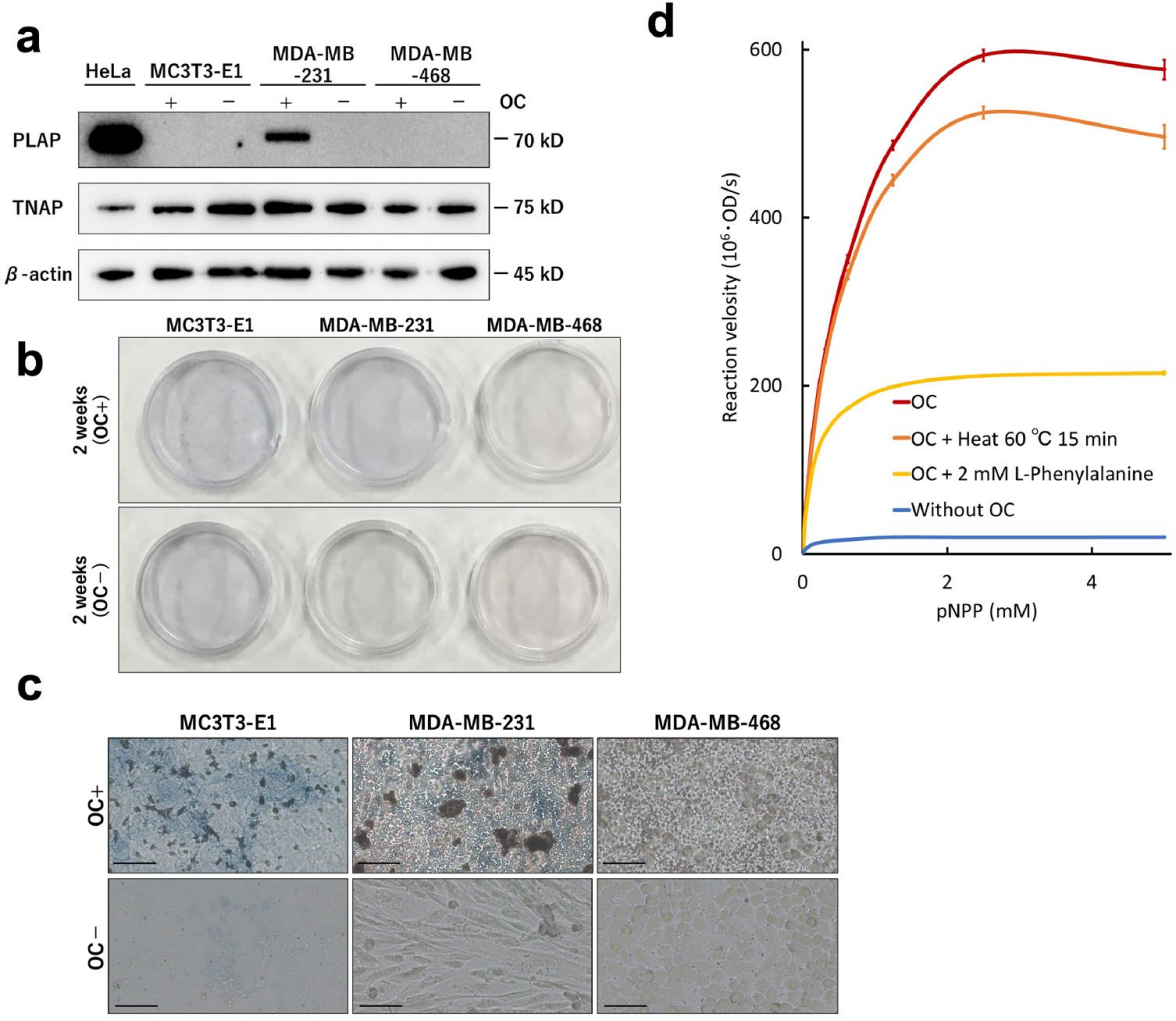
Cultured with an osteogenic cocktail (OC), MDA-MB-231 cells have the expression and enzyme activity of placental alkaline phosphatase (PLAP) MC3T3-E1, MDA-MB-231, and MDA-MB-468 cells which were cultured with and without OC for 2 weeks. (**a**) Western blot for PLAP, tissue-nonspecific alkaline phosphatase (TNAP), and β-actin. (**b**,**c**) ALP indicating ALP enzyme activity (c: magnification × 20, scale bar 10 µm). (**d**) Pharmacologic inhibition of ALP enzyme activity in MDA-MB-231 cells. Red line: with OC, orange: with OC + heat treatment (60 °C for 15 min), yellow: with OC + 2 mM L-phenylalanine and blue: without OC. OD: optical density, pNPP: Para-nitrophenyl phosphate.

Western blot showed that PLAP expression in MDA-MB-231 cells cultured with OC (Fig. 3a). In addition, in all cell lines, TNAP was expressed regardless of the presence or absence of OC (Fig. 3a). Alkaline phosphatase (ALP) staining was performed to confirm ALP enzyme activity. After 2 weeks of culture with OC, MDA-MB-468 cells did not stain for ALP, but MDA-MB-231 cells were blue-stained similarly to MC3T3-E1 cells (Fig. 3b,c). Therefore, in MDA-MB-231 cells, PLAP or TNAP expression was activated. We measured ALP enzyme activity after heat block and pharmacologic inhibition based on the difference between PLAP and TNAP^10^. The ALP enzyme activity of MDA-MB-231 cells in the presence of OC was increased, and the activity was retained with heat treatment at 60 °C. The enzyme activity was inhibited by L-phenylalanine. Since PLAP is resistant to heat denaturation and is inhibited by L-phenylalanine, it was shown that addition of OC to MDA-MB-231 cells resulted in PLAP enzyme activity stronger than that for TNAP.

**Figure 4:**
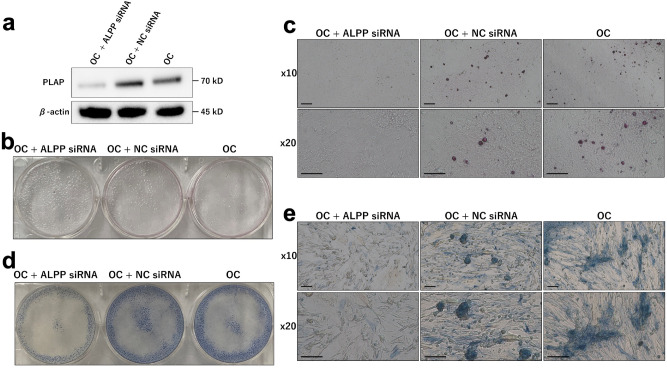
*ALPP* knock-down MDA-MB-231 cells cultured with an osteogenic cocktail (OC) do not show placental alkaline phosphatase (PLAP) expression, calcifications, and alkaline phosphatase (ALP) enzyme activity MDA-MB-231 cells were cultured for 6 weeks with OC including an *ALPP* siRNA or a negative control of OC alone (**a**) Western blot for PLAP after culture for 2 weeks. (**b**,**c**) Alizarin staining shows calcifications (c: magnification × 10 and × 20, scale bar 10 µl). (**d**,**e**) ALP staining shows ALP enzyme activity (e: magnification × 10 and × 20, scale bar 10 µm).

SiRNA assays were performed to determine if PLAP is required for formation of calcifications in MDA-MB-231 cells. First, we confirmed that the PLAP protein expression was decreased when the cells were cultured with OC and ALPP siRNA for 2 weeks (Fig. 4a). After 6 weeks of incubation with OC and ALPP siRNA, no calcifications were seen on alizarin staining (Fig. 4b,c), and ALP enzyme activity was weakened as demonstrated by ALP staining (Fig. 4d,e). Moreover, MDA-MB-468 cells with vector-based enforced expression of PLAP induced calcification when they were cultured with OC for 6 weeks (Supplementary Fig. 2). Therefore, breast cancer cells including MDA-MB-231 cells can induce calcification through PLAP enzyme activity.

**Figure 5:**
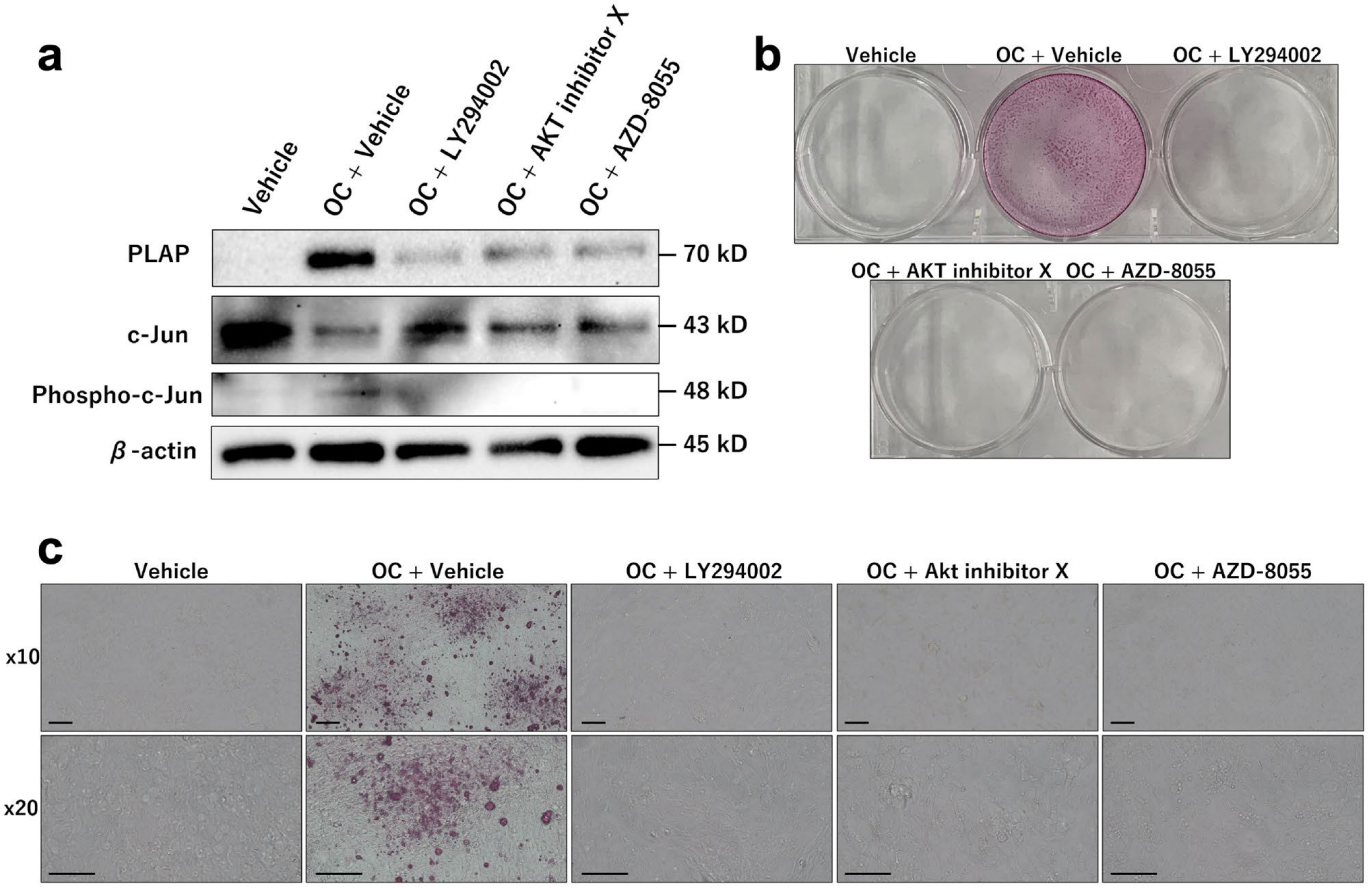
Pharmacologically inhibited PI3K-Akt pathway, MDA-MB-231 cells cultured with an osteogenic cocktail (OC) do not show placental alkaline phosphatase (PLAP) expression and production of calcifications MDA-MB-231 cells were cultured for 6 weeks with vehicle, OC + vehicle, OC + LY294002, OC + Akt inhibitor X, and OC + AZD-8055. (**a**) Western blot for placental alkaline phosphatase (PLAP), c-Jun, and phospho-c-Jun after culture for 2 weeks. (**b**,**c**) Alizarin staining shows calcifications only with OC + vehicle (**c**: magnification × 10 and × 20, scale bar 10 µm).

As microarray analysis indicated that the PI3K-Akt signaling pathway was associated with calcifications in MDA-MB-231 cells, we inhibited the PI3K-Akt signaling pathway in MDA-MB-231 cells cultured using OC. PLAP expression and calcification formation were suppressed by PI3K-Akt signaling pathway inhibitors, namely, LY294002, AKT inhibitor X, and AZD-8055 (Fig. 5). Quantitative reverse transcription PCR (qRT-PCR) showed that the transcription of PLAP was suppressed in MDA-MB-231 cells cultured with OC + Akt inhibitor X and OC + AZD-8055 compared with that in MDA-MB-231 cells cultured with OC only (Supplementary Fig. 3a). Transcription of PLAP in MDA-MB-231 cells cultured with OC + LY294002 was suppressed depending on LY294002 concentration (Supplementary Fig. 3b). RNA-seq in a previous study revealed that c-Jun binds to the promoter region of ALPP in MDA-MB-231 cells^11^. Phospho-c-Jun expression level increased following the addition of OC inhibitors, whereas c-Jun expression level decreased following the addition of OC (Fig. 5a). The results suggest that OC induces c-Jun phosphorylation, which is associated with the PI3K-Akt signaling pathway, and that PLAP transcription would be activated when phospho-c-Jun enters the nucleus and binds to the promoter region of PLAP.

## Discussion

Human breast cancer cell line MDA-MB-231 produced calcifications when cultured with OC, which is used as a standard reagent for osteogenic differentiation from preosteoblast cells in the present study. Interfering RNA technology unveiled that PLAP is as essential for MDA-MB-231 cells to produce calcifications. Furthermore, we found that PI3K-Akt signaling pathway plays an important role in PLAP expression and MDA-MB-231 calcification induction when cultured with OC.

OC is often used in experiments to induce bone differentiation^12–15^. Jaiswal et al. showed that the most appropriate concentrations for bone differentiation are 100 nM of dexamethasone, 0.05 nM of L-ascorbic acid, and 10 mM of β-glycerophosphate^16^. Hamidauche et al. demonstrated that dexamethasone induces mesenchymal cell differentiation when FHL2 is upregulated by attaching dexamethasone to the promoter of the LIM-domain protein with 4.5 LIM domains (FHL2), and it activates WNT/β-catenin signaling-dependent Runx2^17^. Ascorbic acid primarily promotes the formation of collagen, which is the basis of calcification^18^. β-Glycerophosphate is a source of phosphate. In any case, calcification can be induced in osteoblasts simply by supplying these three factors. In this study, we showed that calcifications also occur in breast cancer cell line when cultured in a medium containing OC.

Human breast cancer cell lines have been cultured with several factors to produce calcifications. Cox et al. reported that calcifications in a more invasive subclone of human breast cancer cell line Hs578T using a similar OC^7^. Dang et al. demonstrated production of calcifications in the human breast cancer cell lines MCF7 and Hs578T using ascorbic acid, dexamethasone, and inorganic phosphate rather than β-glycerophosphate^19^. Although calcifications can therefore be induced in breast cancer cells in the presence of inorganic phosphorus, it has been unclear whether it occurs without an inorganic phosphorus in the presence of a phosphoric ester.

ALP enzyme activity is essential to convert phosphoric ester to inorganic phosphorus, allowing utilization of phosphorus in vivo. Hypophosphatasia, which is deficient of ALP, patients have bone fragility^20^ and high levels of phosphomonoester, such as pyridoxal 5′-phosphate and inorganic pyrophosphate^21^. ALP is activated during the process of osteoblast mineralization^22,23^. In the same way, it is presumed that phosphoric ester is the source of phosphorus in breast cancer calcifications and ALP is locally activated on the cancer cells^24^. In fact, O’Grady et al*.* showed that ALP enzyme activity was increased by OC in MDA-MB-231 and SKBR3 cells with calcium deposition^25^. However, the study did not completely unveil how breast cancer cells acquire ALP enzyme activity, although they showed that ALP inhibitor levamisole suppress mineralization in MDA-MB-231 cells cultured using OC.

Our results suggest that PLAP produces ALP enzyme activity required for calcification in MDA-MB-231 cells. PLAP is clinically expressed in some breast cancers^26^. Chang et al*.* showed that dexamethasone induces PLAP enzyme activity and mRNA in the human breast cancer BC-M1 cell line^27^. Therefore, dexamethasone in OC may be associated with PLAP expression in the MDA-MB-231 cells. On the other hand, c-Jun binds to the promoter region of ALPP in MDA-MB-231^11^^,^ and we showed that c-Jun phosphorylation appears in MDA-MB-231 cells cultured using OC (Fig. 5). This may explain why MDA-MB-468 cells did not express PLAP and did not produce calcifications using OC because c-Jun expression of MDA-MB-468 cells is lower than that of MDA-MB-231 cells^28^. We suggest that PLAP transcript would be activated when phospho-c-Jun enters the nucleus and binds to the promoter region of PLAP.

The precise pathway leading to PLAP expression; however, has not yet been reported. In this study, we found that the PI3K-Akt signaling pathway was active in both the MC3T3-E1 osteoblast and the MDA-MB-231 human breast cancer cell lines. Culturing the cells with inhibitors of this pathway decreased PLAP expression and c-Jun phosphorylation, and the cells failed to produce calcifications. The PI3K-Akt signaling pathway plays an important role in cell proliferation and survival and in angiogenesis. Mutations in this pathway are common in breast cancer^29^. Furthermore, calcifications in blood vessels are triggered by increased apoptosis, a cellular event associated with the PI3K-Akt signaling pathway^30–33^. We therefore speculate that the PI3K-Akt signaling pathway may also be involved in calcifications in breast cancer.

Our proposed mechanism in breast calcifications is that OC induces c-Jun phosphorylation via PI3K-Akt signaling pathway and that PLAP transcription would be activated when phospho-c-Jun enters the nucleus and binds to the promoter region of PLAP. Future research might assess whether increased PLAP levels are the main driver of intracellular calcifications in breast cancer, as well as whether the PI3K-Akt signaling pathway has a similar effect in vivo that we demonstrated to cause calcifications in vitro.

## Materials and methods

### Cell culture and reagents

Two human breast cancer cell lines, MDA-MB-231 and MDA-MB-468 and the mouse preosteoblast MC3T3-E1 cell line were provided by ATCC. The cells were cultured with alpha-modified minimum essential medium and 10% fetal bovine serum (Sigma-Aldrich, MO, USA), the latter prepared by heat inactivation. HeLa cells, provided by ATCC, were cultured using Dulbecco’s modified Eagle Medium and 10% fetal bovine serum. Culture of all cell lines was carried out in a 5% CO_2_ air incubator at 37 °C. The medium was changed twice a week. OC was an osteoblast-inducer reagent (Takara Bio. Shiga, Japan) consisting of ascorbic acid 1% (v/v), hydrocortisone 0.2% (v/v), and β-glycerophosphate 2% (v/v).

### Alizarin and ALP staining

For alizarin staining, we fixed the cells for 20 min in ice-cold 100% methanol and then stained with alizarin red using a Calcified Nodule Staining kit (Cosmo Bio, Tokyo, Japan). For ALP staining, we fixed the cells for 20 min in 4% paraformaldehyde and used an alkaline phosphatase staining kit (Cosmo Bio).

### Elemental analysis

For electron microscopic observation, the specimens stained by alizarin were refixed with 2% glutaraldehyde in 0.1 M phosphate buffer overnight at 4 °C. Dehydration was carried out using graded concentrations of ethanol, then the specimens were placed in propylene oxide and subsequently embedded in Epok 812 (Oken, Tokyo, Japan). The specimens were examined with a TM4000 (Hitachi, Tokyo, Japan) electron microscope at 15 kV to analyze their elemental composition.

### Microarray analysis

MDA-MB-231, MDA-MB-468, and MC3T3-E1 cells were seeded into 35-mm dishes at a cell density of 2.0 × 10^5^ cells/ml for 3 days. The cells were cultured with or without OC for 2, 4, and 6 weeks. Total RNA was extracted using TRIzol Reagent (Thermo Fisher Scientific, MA, USA). We measured the RNA integrity number of total RNA using an Agilent RNA 6000 Nano Kit (Agilent technologies, CA, USA) to verify the quality of total RNA. Microarray analysis was performed using Agilent SureScan G4900DA (Agilent technologies).

MA plots were reported from normalized microarray data. We defined Log_2_FC > 1 as upregulated genes and Log_2_FC ≤ 1 as downregulated genes. Pathway analysis of differentially expressed genes was performed using genes up- or downregulated at 2, 4, and 6 weeks. Frequent pathways were extracted in the KEGG pathway from functional analysis of The Database for Annotation, Visualization and Integrated Discovery https://david.ncifcrf.gov)^34,35^. The threshold of count was 2 and the Expression Analysis Systematic Explorer score was 0.1. Gene set enrichment analysis was performed using Qlucore Omics Explorer version 3.4 (Qlucore AB, Lund, Sweden).

### Western blotting

MDA-MB-231, MDA-MB-468, and MC3T3-E1 cells treated were cultured as described above with or without OC for 2 weeks. The cells were then lysed with CHAPS buffer (Cell Signaling Technology, MA, USA). As a positive control for PLAP and TNAP, HeLa cells were cultured for 3 days and their protein extracted. The cell lysates were heated at 100 °C for 5 min with SDS sample buffer, and 10 µg of protein was electrophoresed in 10% acrylamide gel at 150 V for 1 h. The gel was blotted to a cellulose nitrate membrane at 150 mA. Anti-PLAP antibody (EPR6141, Abcam, Cambridge, UK) diluted 1,000 times, anti-TNAP antibody (EPR4477, Abcam) diluted 4,000 times, anti-c-Jun antibody (9165S, Cell Signaling Technology; diluted 2,000 times), anti-phospho-c-Jun antibody (3270S, Cell Signaling Technology; diluted 2,000 times), and anti-β-actin antibody (8H10D10, Cell Signaling Technology) were used as the primary antibodies. Peroxidase AffiniPure goat anti-rabbit IgG (H + L) (111-035-144, Jackson ImmunoResearch, West Grove, PA, USA) diluted 2,000 times for PLAP, TNAP, c-Jun, and phospho-c-Jun and anti-mouse IgG horseradish peroxidase (GE Healthcare, IL, USA) diluted 1,000 times for β-actin were used as secondary antibodies. After adding Thermo Pierce Western Blotting Substrate (Thermo Fisher Scientific), images were acquired with chemiluminescence detection using ChemiDoc Touch (Bio-Rad, CA, USA).

### ALP enzyme kinetics by pharmacologic inhibition

MDA-MB-231 cells were seeded into 96-well plates at a cell density of 2.0 × 10^5^ cells/ml for 3 days. Cells were cultured with and without OC. The cells were fixed for 20 min in 4% paraformaldehyde. The cells cultured with OC were heated at 60 °C for 15 min with and without the addition of 2 mM L-phenylalanine. Para-nitrophenyl phosphate (pNPP) was added at concentrations of 0.01–5 mM. The solution was examined at 405 nm for 30 min every one minute using a microplate reader (Bio-Rad). Michaelis–Menten plots were generated based on the initial reaction velocities that were calculated at each concentration in each group.

### siRNA assay

ALPP siRNA (*Silencer*™ Select Pre-designed & Validated siRNA; s1300 and s1301, Thermo Fisher Scientific) and negative control siRNA (*Silencer*™ Select Negative Control No. 1 siRNA, Thermo Fisher Scientific) were added 1:1 to Lipofectamine RNAiMAX (Thermo Fisher Scientific). MDA-MB-231 was seeded into 6-well plates at a cell density of 2.0 × 10^5^ cells/ml for 3 days. Cells were cultured after addition of OC, OC with siRNA 10 µM (s1300 + s1301), or OC with negative control siRNA 10 µM. We performed western blot after 2 weeks and alizarin and ALP staining after 6 weeks.

### Pharmacologic inhibition for PI3K-Akt signaling pathway

LY294002 (FUJIFILM Wako Pure Chemical, Osaka, Japan) 20 µM, AKT inhibitor X (Cayman Chemical, MI, USA) 20 µM, and AZD-8055 (ChemScene, NJ, USA) 1 µM were added to medium, OC, and vehicle (0.04% DMSO). MDA-MB-231 cells were seeded into 6-well plates at a cell density of 2.0 × 10^5^ cells/ml for 3 days. The cells were with vehicle alone or with OC with added vehicle, LY294002, AKT inhibitor X, or AZD-8055. We performed western blot at 2 weeks and alizarin staining at 6 weeks.

### qRT-PCR

Total cellular RNA was isolated using the TRIzol Reagent (Thermo Fisher Scientific). cDNAs were synthesized using the High Capacity cDNA Reverse Transcription Kit (Applied Biosystems, Grand Island, NY, USA). cDNA samples were amplified using the Power SYBR Green PCR Master Mix (Applied Biosystems) and CFX96 Real-Time PCR System (Bio-Rad). Primers used for qRT-PCR are listed in Supplementary Table 2.

### Vector-based enforced expression

ALPP insert was purified from cDNAs that were synthesized from total cellular RNA in HeLa cells using PrimeSTAR (Takara Bio) and the primers of ALPP (Supplementary Table 2). The insert was inserted into the multiple cloning site of the pcDNA3.1( +) plasmid using the pBlueScript II SK( +) plasmid. Next, MDA-MB-468 cells were transfected with pcDNA3.1( +)–ALPP by electroporation (Bio-Rad) and selected for growth in 300 ug/l geneticin (Cosmo Bio).

### Statistical analysis

Student’s *t*-test was used to determine differences between means of groups. *P* < 0.05 denoted statistical significance. All statistical analyses were performed using R version 3.6.3.

## Supplementary information

Supplementary file1.
