# Supplementary material for: Osteogenic cocktail induces calcifications in human breast cancer cell line via placental alkaline phosphatase expression
Source: Sci Rep. 2020 Jul 29;10:12669. doi: 10.1038/s41598-020-69622-7 (PMC7391638; doi:10.1038/s41598-020-69622-7)

SUPPLEMENTARY INFORMATION

Osteogenic cocktail induces calcifications in human breast cancer cell line via placental alkaline phosphatase expression

Atsushi Fushimi[1,2], Hiroshi Takeyama[2], Toshiaki Tachibana[1], and Yoshinobu Manome[1]

[1]Core Research Facilities, The Jikei University School of Medicine, Tokyo, Japan

[2]Department of Surgery, The Jikei University School of Medicine, Tokyo, Japan

Correspondence to: Atsushi Fushimi, Core Research Facilities, The Jikei University School of Medicine, 3-25-8, Nishi-shimbashi, Minato-ku, Tokyo, Japan. Tel.: +81 334331111, Fax: +81 354001387, e-mail: fushimi@jikei.ac.jp

Supplementary Table 1. Details of elemental analysis of calcification in MDA-MB-231 cells (HITACHI TM4000 HV 15.0, keV 1000×, WD 9.4 mm**)**

Element	Atomic number	Net count	Mass (%)	Atomic concentrati on (%)	Absolute error (1σ) (%)	Relative error (1σ) (%)
C	6	216696	55.09	64.18	6.01	10.90
N	7	5237	6.14	6.14	0.98	15.93
O	8	70889	29.32	25.64	3.40	11.59
Na	11	17163	1.27	0.77	0.11	8.29
P	15	81628	3.63	1.64	0.16	4.51
Cl	17	17359	0.93	0.37	0.06	6.14
Ca	20	43473	3.62	1.26	0.13	3.71

Supplementary Figure 1. Gene set enrichment analysis of MDA-MB-231 cells cultured with or without the osteogenic cocktail. Microarray data shows the findings of gene set enrichment analysis of (a) PI3K-AKT signaling in cancer and (b) mTOR signaling and the heatmaps of (c) PI3K/AKT signaling in cancer and (d) mTOR signaling.

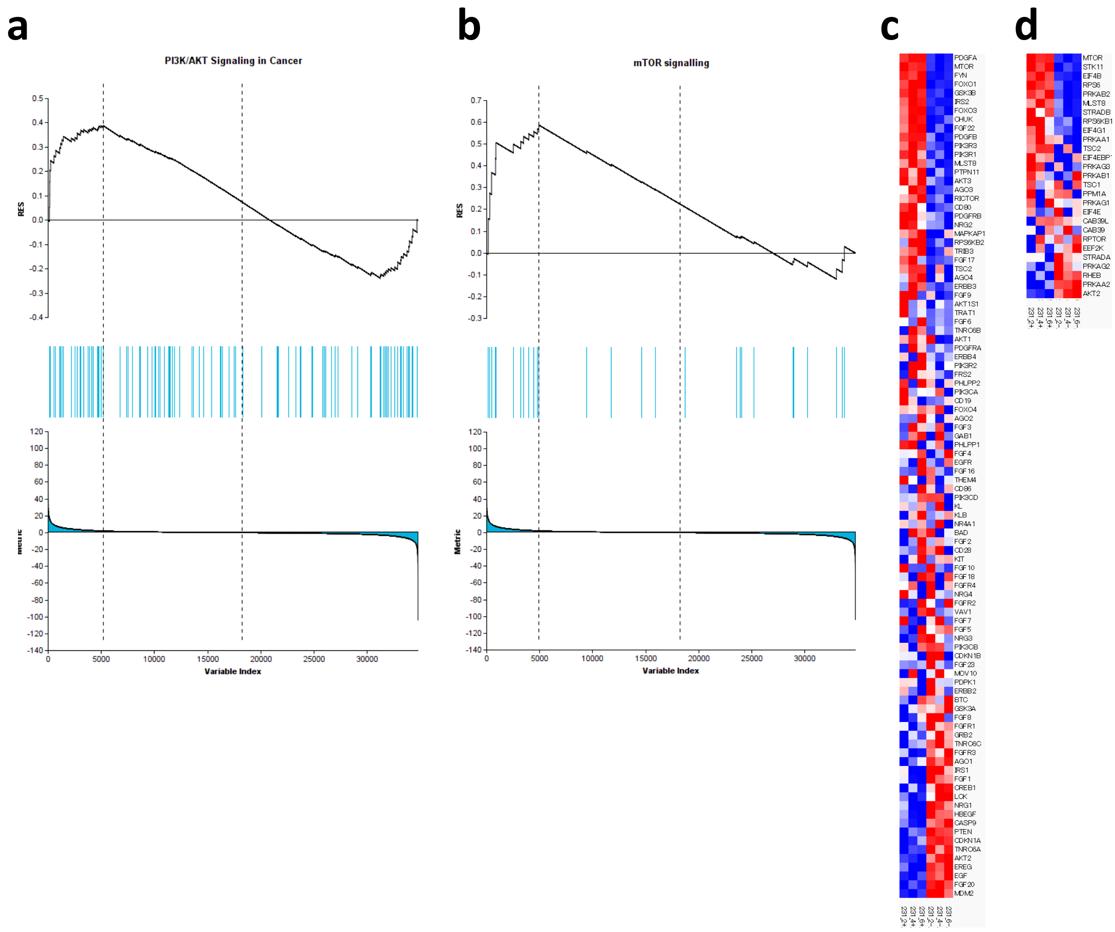

Supplementary Figure 2. Vector-based enforced expression of PLAP in MDA-MB-468 cells induced calcifications when cultured with the osteogenic cocktail (OC). (a) PLAP expression was confirmed on MDA-MB-468 cells based on its vector-based enforced expression using western blots. (b, c) Alizarin staining shows calcification induced by PLAP-expressed MDA-MB-468 cells cultured with OC for 6 weeks (c: magnification ×20, scale bar 10 μm).

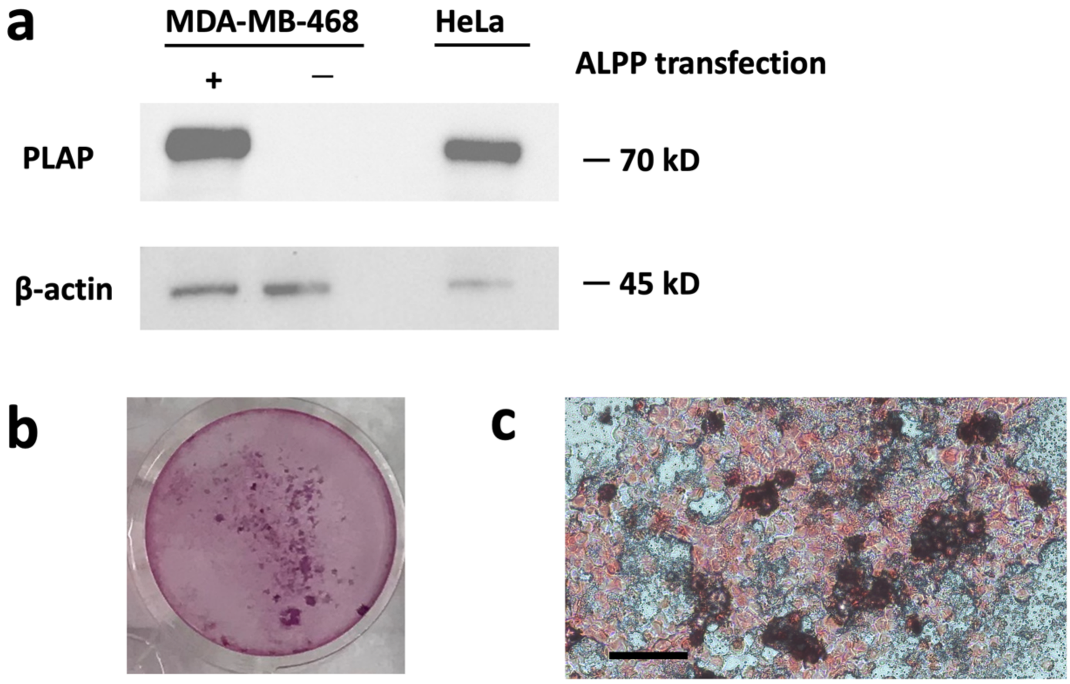

Supplementary Figure 3. PLAP mRNA in MDA-MB-231 cells pharmacologically inhibited the PI3K-Akt pathway when cultured with the osteogenic cocktail (OC). (a) MDA-MB-231 cells were cultured for 7 days with vehicle, OC+vehicle, OC+Akt inhibitor X, and OC+AZD-8055. Two or three asterisks indicate P value less than 0.01 or 0.001, respectively. (b) MDA-MB-231 cells were cultured for 7 days with vehicle, OC+vehicle, and OC+LY294002 (20, 40, and 60 uM). One asterisk indicates P value less than 0.05.

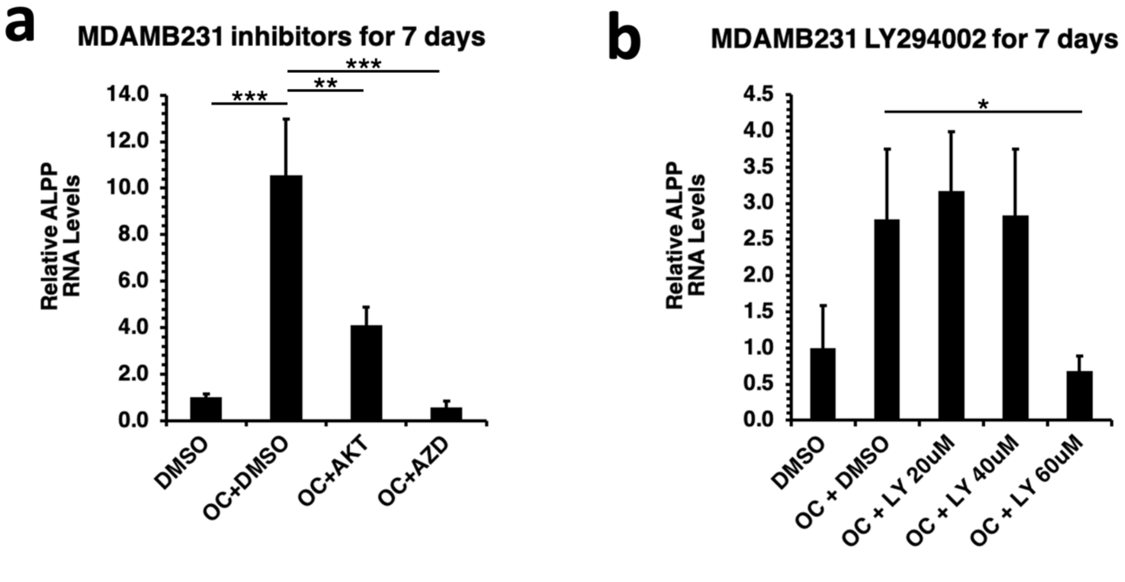

Supplementary Table 2. List of primers used for qRT-PCR and cloning

Primer	Forward (5′–3′)	Reverse (5′–3′)
GAPDH	CCCAGCAAGAGCACAAGAG	CCTCTTCAAGGGGTCTACATG
ALPP<br><br>for qRT-PCR	GACTACAGCCAAGGTGGGAC	GACCTTTGGCTCTCGACCAG
ALPP<br><br>for cloning	CCAGACATGCTGGGGCCCTGCATGCTGCTG	CCCAGGGACGGGACACTCAGGGAGCAGTGG

Figure 3a - PLAP

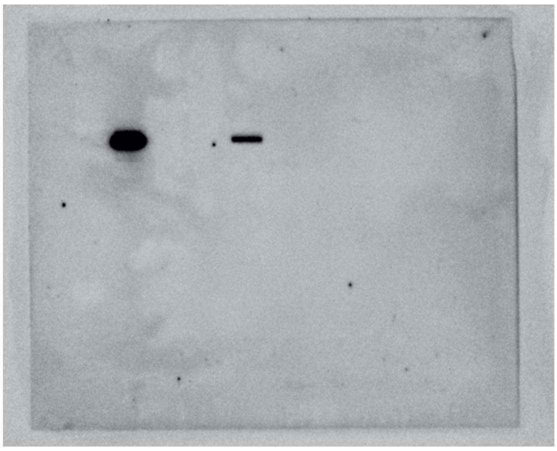

Figure 3a - TNAP

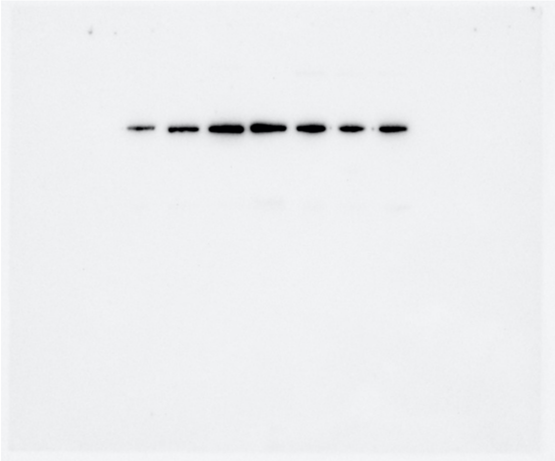

Figure 3a - β-actin

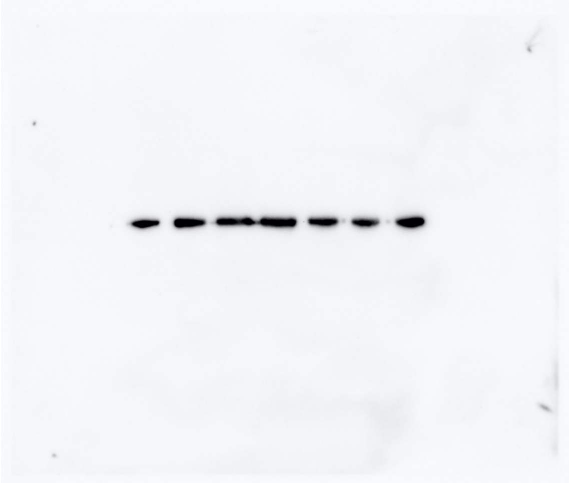

Figure 4a - PLAP

Figure 4a - β-actin

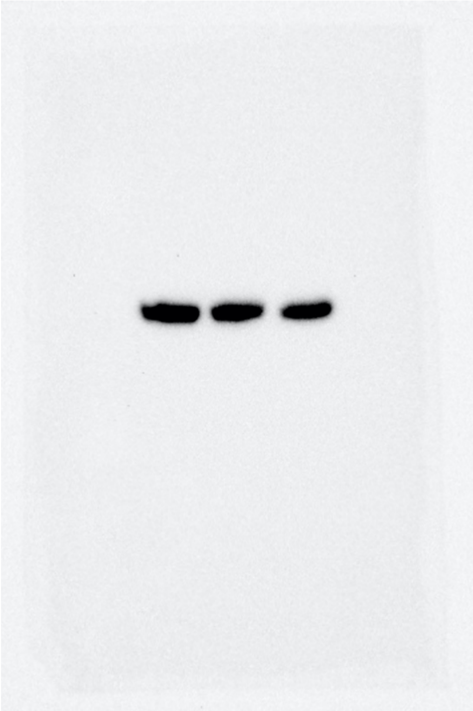

Figure 5a - PLAP

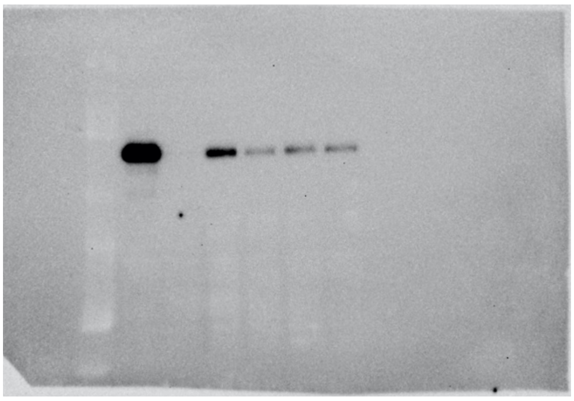

Figure 5a - β -actin

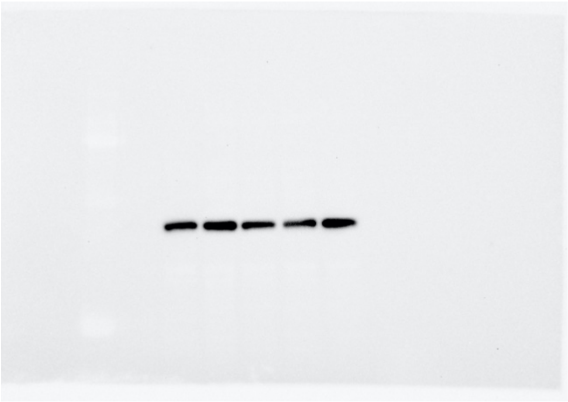

Figure 5a – c-Jun

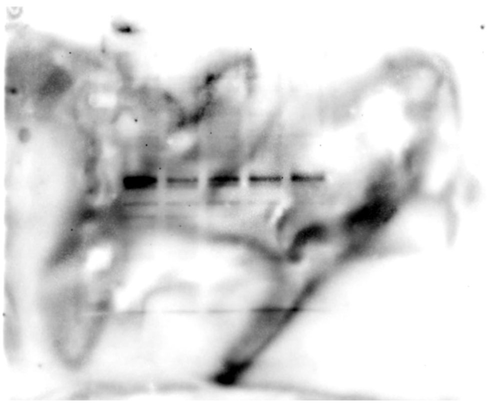

Supplementary Figure 2a - PLAP

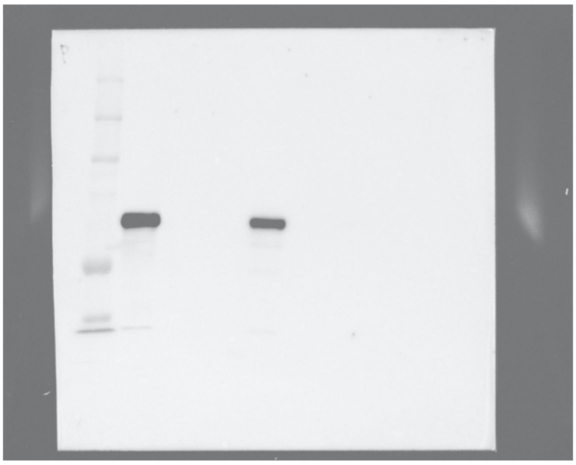

Figure 5a – Phospho-c-Jun

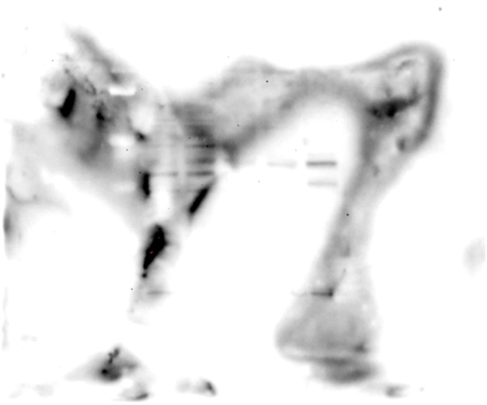

Supplementary Figure 2a - β -actin

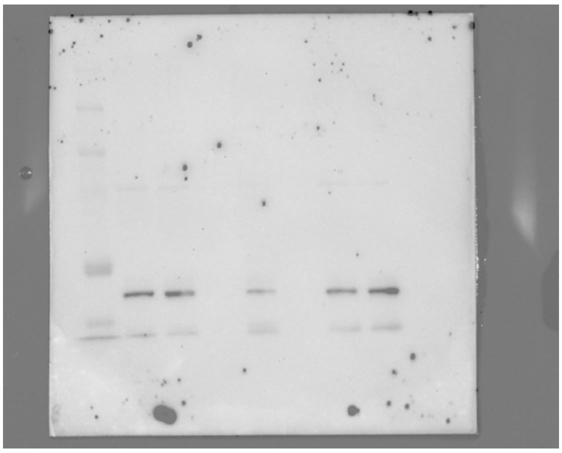

Supplement: Supplementary file 1 — Supplementary file1. [file 41598_2020_69622_MOESM1_ESM.pdf]
